# Supplementary material for: Modeling glioblastoma heterogeneity as a dynamic network of cell states
Source: Mol Syst Biol. 2021 Sep 16;17(9):e10105. doi: 10.15252/msb.202010105 (PMC8444284; doi:10.15252/msb.202010105)
Supplement: Supplementary file 6 — Source Data for Figure 5 [file MSB-17-e10105-s004.zip › Figure5A_sourcedata/GSEA_3071/nosigdb_stateIV.GseaPreranked.1622795164626/RICHARDS_VERHAAK_CANCERCELL_2010_PRONEURAL.html]

Details for gene set RICHARDS\_VERHAAK\_CANCERCELL\_2010\_PRONEURAL[GSEA]

|  || Dataset | state53071 |
| Phenotype | NoPhenotypeAvailable |
| Upregulated in class | na\_pos |
| GeneSet | RICHARDS\_VERHAAK\_CANCERCELL\_2010\_PRONEURAL |
| Enrichment Score (ES) | 0.5795504 |
| Normalized Enrichment Score (NES) | 3.1040099 |
| Nominal p-value | 0.0 |
| FDR q-value | 0.0 |
| FWER p-Value | 0.0 |
Table: GSEA Results Summary

  

Fig 1: Enrichment plot: RICHARDS\_VERHAAK\_CANCERCELL\_2010\_PRONEURAL      
 Profile of the Running ES Score & Positions of GeneSet Members on the Rank Ordered List

  

| PROBE | GENE SYMBOL | GENE\_TITLE | RANK IN GENE LIST | RANK METRIC SCORE | RUNNING ES | CORE ENRICHMENT || 1 | C1orf61 |  |  | 3 | 2.760 | 0.0343 | Yes |
| 2 | DCX |  |  | 4 | 2.752 | 0.0691 | Yes |
| 3 | DGKI |  |  | 13 | 2.442 | 0.0984 | Yes |
| 4 | BEX1 |  |  | 18 | 2.252 | 0.1261 | Yes |
| 5 | FXYD6 |  |  | 19 | 2.252 | 0.1546 | Yes |
| 6 | SLCO5A1 |  |  | 35 | 1.977 | 0.1767 | Yes |
| 7 | ASCL1 |  |  | 53 | 1.813 | 0.1964 | Yes |
| 8 | MAP2 |  |  | 55 | 1.811 | 0.2191 | Yes |
| 9 | SOX11 |  |  | 56 | 1.811 | 0.2419 | Yes |
| 10 | CKB |  |  | 72 | 1.711 | 0.2607 | Yes |
| 11 | NRXN2 |  |  | 83 | 1.621 | 0.2793 | Yes |
| 12 | MMP16 |  |  | 90 | 1.578 | 0.2981 | Yes |
| 13 | GPM6A |  |  | 98 | 1.538 | 0.3162 | Yes |
| 14 | TMCC1 |  |  | 102 | 1.524 | 0.3348 | Yes |
| 15 | DLL3 |  |  | 105 | 1.521 | 0.3537 | Yes |
| 16 | MYO10 |  |  | 121 | 1.484 | 0.3696 | Yes |
| 17 | NCAM1 |  |  | 129 | 1.456 | 0.3866 | Yes |
| 18 | CHD7 |  |  | 144 | 1.386 | 0.4014 | Yes |
| 19 | GRIA2 |  |  | 160 | 1.347 | 0.4156 | Yes |
| 20 | ZEB2 |  |  | 164 | 1.339 | 0.4319 | Yes |
| 21 | C1QL1 |  |  | 195 | 1.272 | 0.4422 | Yes |
| 22 | SOX2 |  |  | 212 | 1.231 | 0.4547 | Yes |
| 23 | SATB1 |  |  | 222 | 1.203 | 0.4682 | Yes |
| 24 | ERBB3 |  |  | 243 | 1.164 | 0.4791 | Yes |
| 25 | FHOD3 |  |  | 245 | 1.161 | 0.4936 | Yes |
| 26 | MPPED2 |  |  | 267 | 1.108 | 0.5035 | Yes |
| 27 | PFN2 |  |  | 270 | 1.107 | 0.5171 | Yes |
| 28 | PODXL2 |  |  | 326 | 0.992 | 0.5191 | Yes |
| 29 | CDKN1B |  |  | 374 | 0.939 | 0.5219 | Yes |
| 30 | RUFY3 |  |  | 376 | 0.929 | 0.5335 | Yes |
| 31 | SOX4 |  |  | 377 | 0.928 | 0.5452 | Yes |
| 32 | TOP2B |  |  | 382 | 0.924 | 0.5561 | Yes |
| 33 | TTYH1 |  |  | 404 | 0.906 | 0.5635 | Yes |
| 34 | MARCKSL1 |  |  | 417 | 0.888 | 0.5725 | Yes |
| 35 | CRMP1 |  |  | 438 | 0.866 | 0.5796 | Yes |
| 36 | ABAT |  |  | 515 | 0.805 | 0.5751 | No |
| 37 | BCL7A |  |  | 548 | 0.765 | 0.5786 | No |
| 38 | SCG3 |  |  | 632 | 0.707 | 0.5716 | No |
| 39 | ARHGEF9 |  |  | 688 | 0.667 | 0.5695 | No |
| 40 | ZNF711 |  |  | 774 | 0.618 | 0.5609 | No |
| 41 | SRGAP3 |  |  | 789 | 0.609 | 0.5660 | No |
| 42 | MAPT |  |  | 844 | 0.583 | 0.5629 | No |
| 43 | STMN4 |  |  | 876 | 0.570 | 0.5642 | No |
| 44 | PHLPP1 |  |  | 977 | 0.533 | 0.5517 | No |
| 45 | CLASP2 |  |  | 1017 | 0.515 | 0.5507 | No |
| 46 | ZNF286A |  |  | 1020 | 0.514 | 0.5568 | No |
| 47 | PAK3 |  |  | 1046 | 0.506 | 0.5584 | No |
| 48 | TSPAN3 |  |  | 1093 | 0.494 | 0.5558 | No |
| 49 | OLIG2 |  |  | 1129 | 0.486 | 0.5552 | No |
| 50 | GADD45G |  |  | 1147 | 0.481 | 0.5580 | No |
| 51 | LRP6 |  |  | 1162 | 0.477 | 0.5614 | No |
| 52 | YPEL1 |  |  | 1187 | 0.470 | 0.5627 | No |
| 53 | ZNF821 |  |  | 1197 | 0.465 | 0.5668 | No |
| 54 | GSK3B |  |  | 1227 | 0.457 | 0.5670 | No |
| 55 | GNG4 |  |  | 1230 | 0.457 | 0.5724 | No |
| 56 | DCAF7 |  |  | 1251 | 0.452 | 0.5743 | No |
| 57 | FBXO21 |  |  | 1257 | 0.451 | 0.5790 | No |
| 58 | TMEFF1 |  |  | 1310 | 0.438 | 0.5746 | No |
| 59 | ATAT1 |  |  | 1318 | 0.436 | 0.5787 | No |
| 60 | CSNK1E |  |  | 1412 | 0.413 | 0.5661 | No |
| 61 | RNFT2 |  |  | 1426 | 0.410 | 0.5688 | No |
| 62 | FAM110B |  |  | 1440 | 0.405 | 0.5714 | No |
| 63 | IL1RAPL1 |  |  | 1523 | 0.384 | 0.5605 | No |
| 64 | TMSB15A |  |  | 1540 | 0.381 | 0.5622 | No |
| 65 | GSTA4 |  |  | 1702 | 0.349 | 0.5357 | No |
| 66 | MTSS1 |  |  | 1708 | 0.348 | 0.5391 | No |
| 67 | MARCKS |  |  | 1713 | 0.348 | 0.5427 | No |
| 68 | RAB33A |  |  | 1760 | 0.337 | 0.5381 | No |
| 69 | NLGN3 |  |  | 1785 | 0.333 | 0.5377 | No |
| 70 | DBN1 |  |  | 1846 | 0.325 | 0.5303 | No |
| 71 | RAP2A |  |  | 1903 | 0.314 | 0.5235 | No |
| 72 | PELI1 |  |  | 1921 | 0.310 | 0.5242 | No |
| 73 | MLLT11 |  |  | 1932 | 0.308 | 0.5261 | No |
| 74 | ZNF248 |  |  | 2009 | 0.295 | 0.5152 | No |
| 75 | CASK |  |  | 2014 | 0.294 | 0.5182 | No |
| 76 | TOX3 |  |  | 2039 | 0.290 | 0.5173 | No |
| 77 | DPYSL4 |  |  | 2060 | 0.288 | 0.5170 | No |
| 78 | SEZ6L |  |  | 2065 | 0.287 | 0.5199 | No |
| 79 | CDK5R1 |  |  | 2126 | 0.280 | 0.5119 | No |
| 80 | VEZF1 |  |  | 2161 | 0.274 | 0.5088 | No |
| 81 | DUSP26 |  |  | 2260 | 0.260 | 0.4933 | No |
| 82 | MYT1 |  |  | 2292 | 0.257 | 0.4906 | No |
| 83 | CDC25A |  |  | 2753 | -0.301 | 0.4059 | No |
| 84 | FERMT1 |  |  | 4319 | -0.602 | 0.1126 | No |
| 85 | EPB41 |  |  | 4449 | -0.654 | 0.0961 | No |
| 86 | KLRC3 |  |  | 4540 | -0.700 | 0.0877 | No |
| 87 | ADGRL3 |  |  | 4800 | -0.866 | 0.0488 | No |
| 88 | ATAD5 |  |  | 4934 | -0.998 | 0.0358 | No |
| 89 | MCM10 |  |  | 5066 | -1.222 | 0.0261 | No |
| 90 | ALCAM |  |  | 5118 | -1.327 | 0.0331 | No |
Table: GSEA details [plain text format]

  

Fig 2: RICHARDS\_VERHAAK\_CANCERCELL\_2010\_PRONEURAL: Random ES distribution      
 Gene set null distribution of ES for **RICHARDS\_VERHAAK\_CANCERCELL\_2010\_PRONEURAL**
